# RNA-Seq following PCR-based sorting reveals rare cell transcriptional signatures

**DOI:** 10.1186/s12864-016-2694-2

**Published:** 2016-05-17

**Authors:** Maurizio Pellegrino, Adam Sciambi, Jamie L. Yates, Joshua D. Mast, Charles Silver, Dennis J. Eastburn

**Affiliations:** Mission Bio, Inc., 953 Indiana St., San Francisco, California 94107 USA

**Keywords:** Transcriptome, Droplets, Cell sorting, Heterogeneity, Single-cell, Microfluidics, Gene expression, PCR

## Abstract

**Background:**

Rare cell subtypes can profoundly impact the course of human health and disease, yet their presence within a sample is often missed with bulk molecular analysis. Single-cell analysis tools such as FACS, FISH-FC and single-cell barcode-based sequencing can investigate cellular heterogeneity; however, they have significant limitations that impede their ability to identify and transcriptionally characterize many rare cell subpopulations.

**Results:**

PCR-activated cell sorting (PACS) is a novel cytometry method that uses single-cell TaqMan PCR reactions performed in microfluidic droplets to identify and isolate cell subtypes with high-throughput. Here, we extend this method and demonstrate that PACS enables high-dimensional molecular profiling on TaqMan-targeted cells. Using a random priming RNA-Seq strategy, we obtained high-fidelity transcriptome measurements following PACS sorting of prostate cancer cells from a heterogeneous population. The sequencing data revealed prostate cancer gene expression profiles that were obscured in the unsorted populations. Single-cell expression analysis with PACS was subsequently used to confirm a number of the differentially expressed genes identified with RNA sequencing.

**Conclusions:**

PACS requires minimal sample processing, uses readily available TaqMan assays and can isolate cell subtypes with high sensitivity. We have now validated a method for performing next-generation sequencing on mRNA obtained from PACS isolated cells. This capability makes PACS well suited for transcriptional profiling of rare cells from complex populations to obtain maximal biological insight into cell states and behaviors.

**Electronic supplementary material:**

The online version of this article (doi:10.1186/s12864-016-2694-2) contains supplementary material, which is available to authorized users.

## Background

The analysis of rare and biologically important cell subtypes presents a common challenge in the study of cancer, immunology, development and infectious disease. Subtypes within a sample are often not observable through bulk molecular measurements performed on the entire population [1–4]. Consequently, tools that can individually analyze single cells within a population are essential for uncovering critical biological information on subtypes. Fluorescence Activated Cell Sorting (FACS) is one such single-cell analysis method that has been widely employed to characterize heterogeneous populations of cells [5, 6]. Although FACS is very high in throughput and can recover cells for downstream analysis, it relies on antibody staining that can be laborious and is often low in sensitivity. More importantly, antibodies are unable to characterize nucleic acid based biomarkers such as transcripts, genomic DNA, and mRNA splice variants. To overcome this limitation, cytometry methods that rely on Fluorescence *in situ* Hybridization (FISH) have been used to enumerate and sort cells based on nucleic acid sequences of interest [7–9]; however, FISH-flow cytometry requires numerous sample processing steps that can result in significant cell loss, alter the gene expression profile of the cell or preclude downstream sequencing of the isolated cells.

A promising new approach to single-cell analysis relies upon molecular barcodes that are paired with the transcriptomes of individual cells confined to microwells or emulsion droplets [10–12]. The barcoded oligonucleotides enable reverse transcription of polyadenylated mRNAs and are used to reconstruct, *in silico*, the gene expression profiles of individual cells following sequencing of the pooled single-cell RNA-Seq libraries. The relatively unbiased nature of this type of approach makes it a powerful “bottom up” tool for the discovery of unknown cell subtypes [13]. Although barcoding methods improve upon the throughput of previous single-cell sequencing methods [14], most of them are restricted to the analysis of only hundreds to a few thousand cells per experiment and many of the cells within a sample can be lost due to inefficient barcode pairing [12]. This significant throughput limitation makes them unsuitable for biological samples consisting of tens or hundreds of thousands of cells.

We previously introduced a novel cytometry technology, PCR-activated cell sorting (PACS), which is able to analyze more than 100,000 individual cells in parallel [15], a level of throughput over 40-fold higher than single-cell barcode sequencing methodologies. PACS works by interrogating individual cells with multiplexed TaqMan PCR assays performed in microfluidic droplets for the presence of specific combinations of transcripts, splice variants, non-coding RNAs or genomic DNA and accurately sorts the cell material for further processing [15, 16]. The use of readily available TaqMan assays enables PACS to sort cells with high-specificity, low cost and minimal assay optimization-major advantages over other cytometry approaches. Another key feature of PACS is the use of a two-step microfluidic workflow that first compartmentalizes cells into droplets and then prepares the cell lysate for amplification prior to subsequent microfluidic addition of the TaqMan RT-PCR reagents. This approach is critical for mitigating non-specific TaqMan probe fluorescence and inhibition of RT-PCR enzymes caused by high concentrations of untreated crude cell lysate in microdroplets [17–20]. Additionally, this two-step microfluidic workflow affords the use of smaller volume microdroplets that both reduce reagent cost and enable high throughput.

PACS, like FACS and FISH-flow cytometry, is a “top down” approach to subdividing cell populations [13]. This approach requires pre-selection of known nucleic acid biomarkers for the multiplex TaqMan reactions and subsequent cell subtype classification. While the enumeration of cell subtypes provides valuable information to researchers, for PACS to be most useful, it would not only offer a unique and advantageous approach for the initial high-throughput cell classification and enrichment, but also enable unbiased high-dimensional profiling of gene expression following isolation of subtypes. This additional capability would make PACS ideally-suited for the analysis of subtypes from large heterogeneous cell populations such as circulating tumor cells partially enriched from blood [21–23], disaggregated solid tumors [24–28], leukemias [29–32], virally infected cells [33–35], stem cells [36, 37] and subpopulations of the immune system [13, 38, 39].

In this report, we further characterize PACS and show that the method is capable of sensitive detection of prostate cancer cells across multiple TaqMan assays and cell types. We also demonstrate unbiased RNA sequencing on PACS isolated cells and show that this approach can be used to uncover the gene expression profiles of cells that were originally masked by heterogeneity in the unsorted population. These capabilities make PACS valuable for isolating rare and clinically relevant cell subtypes for comprehensive molecular characterization.

## Results

### PACS workflow for expression profiling

**Fig. 1:**
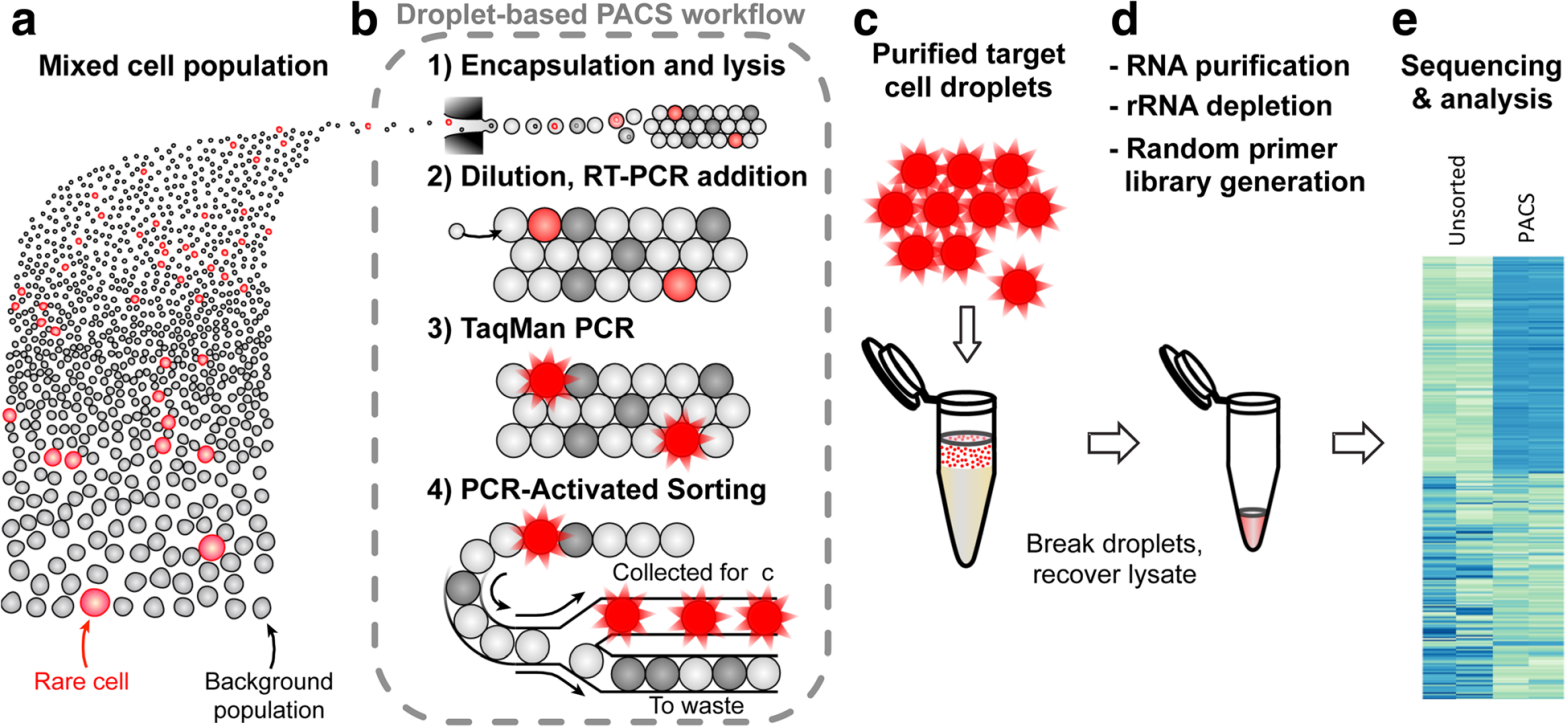
Workflow for droplet-based rare cell enrichment and analysis. **a** Single cells from a heterogeneous population are encapsulated in microfluidic droplets and lysed (**b**). The cell content is merged with RT-PCR reagents, and single cell TaqMan assays are performed in droplets. PCR-Activated Cell Sorting (PACS) allows the recovery of droplets with the desired TaqMan fluorescence profile (**c**). After breaking the emulsion, the nucleic acids are recovered and used for downstream analysis, including library preparation for RNA-Seq (**d**) and gene expression analysis (**e**)

To investigate heterogeneous cell populations with the PACS workflow, cells from a mixed cell suspension are first encapsulated and lysed in microdroplets (Fig. 1a and b). The cells are encapsulated at limiting dilution such that most drops are empty but ~1–5 % contain single cells, in a process governed by Poisson statistics. Following cell lysis and thermal incubation, the droplet-compartmentalized lysate is then merged with RT-PCR reagents and in-droplet TaqMan PCR assays are performed to identify the presence of target nucleic acids (Fig. 1b). The reactions can be multiplexed to detect the expression of specific combinations of nucleic acids in individual cells by using separate TaqMan hydrolysis probes, each linked to a different fluorophore. The TaqMan generated fluorescence values associated with single cells in droplets can be visualized on scatter plots and used to trigger dielectrophoretic droplet sorting when the desired target subtype is identified (Fig. 1b) [15, 40, 41]. The cell contents from the isolated droplets are then recovered and prepared for downstream molecular analysis, including transcriptome profiling (Fig. 1c-e). Unlike our previous method that sampled a portion of each cell’s lysate for single-cell droplet PCR [16], the totality of the lysate is now analyzed and sorted with PACS.

### Sensitive rare cell detection with PACS

**Fig. 2:**
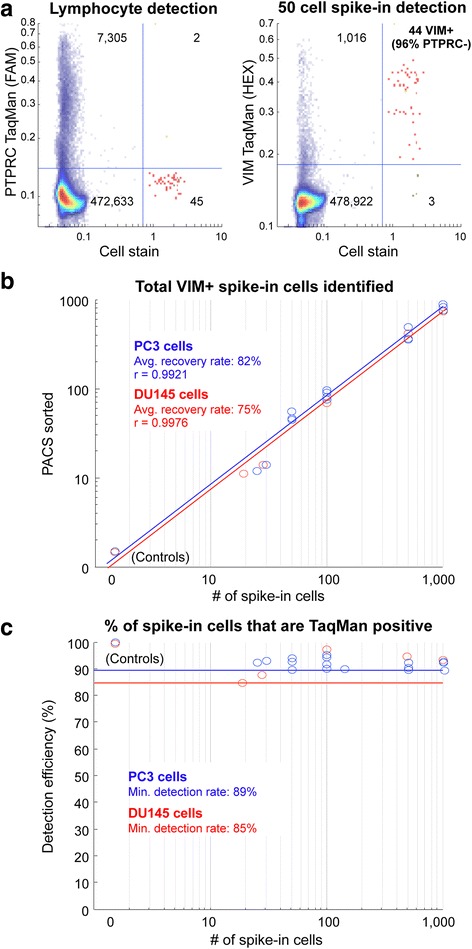
Sensitivity and specificity of detection of rare cells using PACS. **a** Scatter-plot diagram of cell stain intensity values, and PTPRC (FAM, lymphocyte staining, left) or VIM (HEX, PC3 staining, right) fluorescence from multiplexed TaqMan assays. 44 out of 47 PC3 cells expressed VIM, and only 2 of 44 also expressed PTPRC. The blue lines represent the thresholds applied to differentiate clusters; the heat map correlates with drop counts. **b** Dynamic range of PACS. Different numbers of PC3 (blue circles) or DU145 (red circles) cancer cells were spiked in and sorted from a background of lymphocyte Raji cells. The scatter plot shows strong correlation between the number of cells spiked in and the number of VIM+ drops, with a recovery of 82 % and 75 % for PC3 and DU145, respectively. Straight lines represent the fit across spike-ins. **c** The scatter plot shows the reproducibility of the PACS workflow across spike-ins in the two cancer cell lines. The detection efficiency (number of spiked-in cells that show a positive TaqMan signal) is consistently above 89 % (blue line) and 85 % (red line) for PC3 and DU145 cells, respectively

The analysis of rare cells within a population requires detection that is both specific for the target cells and also capable of sensitively interrogating each and every cell within a sample. Cell spike-in experiments offer a straightforward way to assess PACS detection efficiency on a known number of target cells. To investigate the specificity and sensitivity of our method for target cell spike-in detection, we first established a multiplex TaqMan PCR assay that could be used to precisely identify differing numbers of prostate cancer cells from a background population of lymphocytes. Vimentin (VIM gene) is an intermediate filament protein that is highly expressed in cells undergoing epithelial-to-mesenchimal transitions and is generally absent from cells of lymphopoietic origin [15, 42]; conversely, PTPRC is commonly expressed in leukocytes, but not highly expressed in prostate cell types [16, 43, 44]. A multiplex TaqMan assay targeting VIM and PTPRC transcripts should identify both lymphocyte and prostate cancer cell types used in our spike-in experiments. In the example spike-in assay shown in Fig. 2a, 50 PC3 cells were mixed with ~10,000 Raji cells and analyzed with the PACS workflow using a HEX-labeled TaqMan assay targeting VIM, and a FAM-labeled TaqMan assay targeting PTPRC. To independently verify correlation of VIM+ TaqMan signal with spike-in cells, the prostate cancer cells were stained with calcein violet. As shown in the scatter plots, we efficiently identified a total of 47 out of 50 (94 %) calcein positive spike-in cells with PACS. Furthermore, the majority of the drops containing calcein violet were also positive for VIM expression (HEX signal positive, 44 out of 47–94 %, right panel) confirming the specificity and sensitivity of single-cell TaqMan detection in droplets. 2 of the 44 VIM-positive drops (5 %) were also positive for PTPRC FAM signal. This was likely due to either low-level PTPRC expression in some PC3 cells or co-encapsulation with a background Raji cell. The PTPRC+ droplets were easily excluded from sorting, preventing possible contamination of the sorted cancer cells with undesirable background cells.

To further characterize the sensitivity and limit of cell detection with the PACS workflow, we spiked calcein-labeled PC3 cells into ~10,000 Raji cells at cell numbers of 0, 25, 50, 100, 500 and 1000 cells. Using the VIM+/PTPRC- TaqMan assay selection criteria, we accurately identified and recovered, on average, 82 % of PC3 input cells across the different spike-in cell populations with PACS (Fig. 2b). Notably, VIM+ TaqMan correlation with the calcein positive droplets displayed an average of 92 % and a minimum of 89 % TaqMan detection rates for these experiments (Fig. 2c). Critically, these results were not unique to PC3 cells. We obtained similar PACS results with a different prostate cancer cell line, DU145. On average 75 % of the calcein positive DU145 input spike-ins were identified, with an average VIM+ TaqMan detection rate of 93 % and a minimum of 85 % (Fig. 2c).

We also investigated whether background cell populations that more closely resemble a complex primary biological sample affected PACS detection of prostate cancer cells. Peripheral Blood Mononuclear Cells (PBMCs) were isolated from whole blood and used as the background cell population for PC3 cell spike-in experiments. We and others have observed significant vimentin expression within PBMC cell populations [45]; consequently, we used TaqMan assays targeting EpCAM and ARHGAP29 transcripts for specific identification of prostate cancer cells. Similar to our results shown in Fig. 2a, we identified spiked-in PC3 cells at a sensitivity of 83 % with the TaqMan assay targeting EPCAM and 94 % with the TaqMan assay targeting ARHGAP29 (Additional file 1: Figure S1) [23, 43, 46]. Collectively, these data indicate that PACS is capable of detecting multiple rare cell types with high specificity and sensitivity.

### Rare cell molecular analysis following PACS isolation

**Fig. 3:**
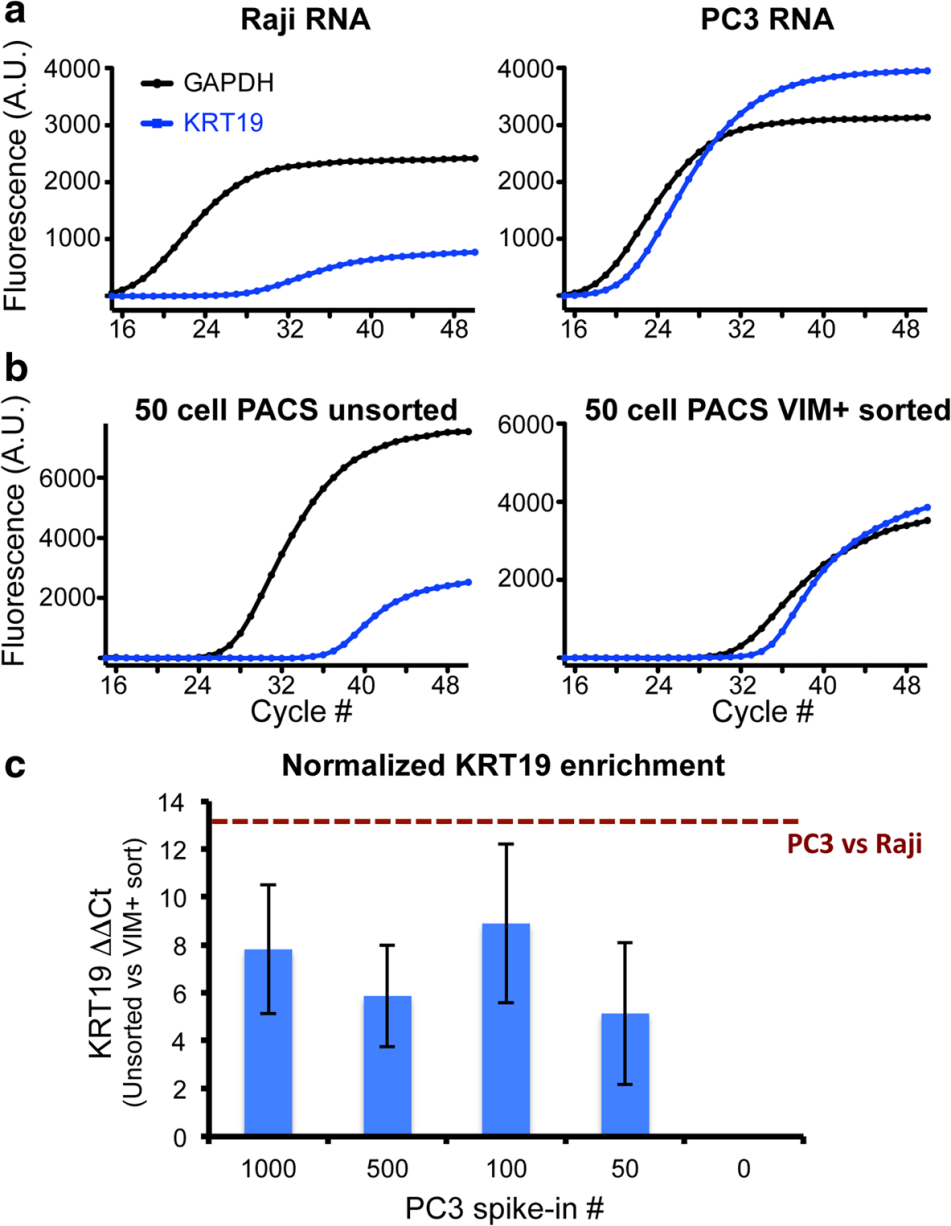
KRT19 expression analysis on PACS-sorted cells. **a**-**b** Representative qRT-PCR amplification curves using RNA from pure Raji and PC3 populations (**a**), or a 50 PC3 cell spike-in unsorted and VIM + −sorted material (**b**) amplified using TaqMan assays targeting GAPDH and the PC3-specific KRT19 gene. **c** Relative quantification of KRT19 in VIM + −sorted lysate compared to material from a heterogeneous population across several cell spike-ins shows reliable enrichment in sorted material. Dark red line represents the maximum ΔΔCt KRT19 calculated as the difference in ΔCt values between pure PC3 and Raji populations. Error bars = SEM

To test whether PACS sorting can efficiently enrich target cell mRNA for downstream analysis, we examined KRT19 transcript levels before and after sorting (Fig. 3). KRT19 is a cytokeratin that is expressed by many epithelial cancer cells but mostly absent in lymphocytes [22, 23]. We quantified the expression of this transcript in the cell lysate derived from both unsorted PC3:Raji cell spike-in populations as well as PACS VIM+/PTPRC- sorted spike-ins (all spike in populations contained 10,000 Raji cells prior to PACS sorting), and compared its relative abundance to GAPDH to control for different amounts of input material. We reasoned that if PACS accurately identified and sorted PC3 cells from the heterogeneous population, we would expect the relative abundance of KRT19/GAPDH following sorting to resemble that of the pure PC3 cells, while the unsorted material would show reduced KRT19 levels due to the presence of mostly Raji cells with a small percentage of high-expressing PC3 cells. Indeed, qRT-PCR analysis on material recovered from a PACS sorted 50 PC3 cell spike-in experiment showed that KRT19 and GAPDH were expressed at similar levels, and closely matched the relative expression observed for the pure PC3 population (Fig. 3a,b). As expected, pre-sorted material had much lower KRT19 expression relative to GAPDH (Fig. 3b). PACS-sorted material shows a similar level of KRT19/GAPDH enrichment across the 100, 500 and 1000 cell spike-in numbers, again validating that our approach is both accurate and sensitive (Fig. 3c). The variability observed in these experiments could be attributed to biological noise. With the small number of cells analyzed, cell-to-cell variability in gene expression can play a substantial role in the overall expression pattern of KRT19. Additionally, effects from RNA loss during processing and handling could also increase with decreasing cell numbers leading to higher variability in the qRT-PCR reactions.

### Transcriptome analysis of PACS-sorted cancer cells

**Fig. 4:**
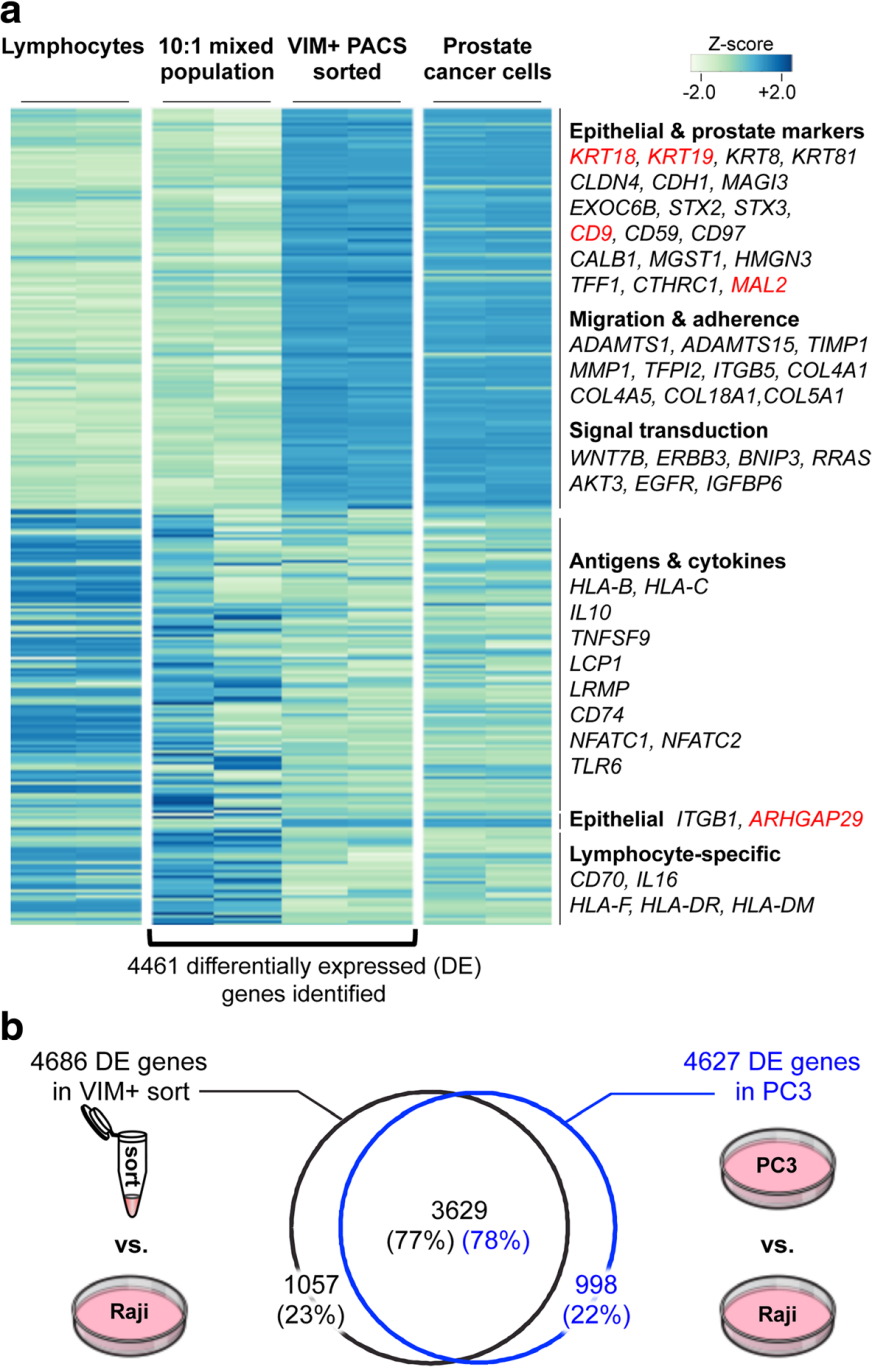
Transcriptome analysis of PACS-sorted cells. **a** Hierarchical clustering of the 4461 genes differentially expressed (DE) between VIM + −sorted droplets and the Raji:PC3 (10:1) starting heterogeneous population. 1,126 and 1,130 VIM+ sorted cells were sequenced from the two replicate experiments. The expression of the same 4461 genes in the pure lymphocyte (Raji) and prostate cancer cell (PC3) populations are shown for comparison. The genes in red were independently verified as being differentially expressed in PC3 cells. The heat map represents Z-scores of log_2_ counts per million (**b**) Venn diagram showing the number of DE genes in common between prostate cancer PC3 cells and VIM + −sorted material when each is independently compared to the lymphocyte Raji cell line. Of the 4686 DE genes in VIM + −sorted material, and 4627 DE genes in PC3 cells, 3629 (77 % and 78 %, respectively) are in common

High-dimensional, quantitative gene expression analysis with RNA sequencing can yield crucial insight into rare cell states and behaviors [13, 14]. The successful enrichment and qRT-PCR analysis of KRT19 expression indicated that next-generation transcriptome sequencing on PACS isolated cell subtypes might be possible. Thermocycling can potentially reduce the amount of intact RNA in a sample; therefore, we chose to optimize a random priming approach for RNA-Seq (see Methods). We generated transcriptome libraries from replicate VIM+/PTPRC- sorted cell lysate derived from 1000 PC3 cells spiked into 10,000 B-lymphocyte Raji cells, and compared them with libraries prepared from unsorted heterogeneous cell spike-in populations (Fig. 4a). Overall, we identified 4,461 differentially expressed genes (FDR ≤ 0.05), 2,242 of which were enriched in the VIM + −sorted population and 2,219 in the starting unsorted population (log_2_-fold change between 1.14 and 12.29, Additional file 2: Figure S2). We then analyzed the 4,461 differentially expressed genes in RNA-Seq data obtained from pure PC3 or Raji RNA (Fig. 4a). Strikingly, the expression profiles from pure PC3 RNA closely resembled the VIM + −sorted RNA (Pearson’s ρ = 0.866, Fig. 4a and Additional file 3: Figure S3a), indicating that PACS successfully enriched PC3 cells and uncovered the prostate cancer cell transcriptional signature that was not observable in the unsorted population. The transcriptional signatures of the unsorted spike-in population and the Raji cells are also well correlated (Pearson’s ρ = 0.796, Fig. 4a and Additional file 3: Figure S3b); however, as expected, the correlation is slightly lower than between the VIM^+^-sorted and PC3 samples due to the presence of PC3 cells in the unsorted population (10:1, Raji:PC3).

We expected the PACS VIM + −sorted material to be enriched in transcripts representative of prostate cells that are epithelial in origin, and to be depleted of genes typical of immune cells. Indeed, genes differentially expressed in the VIM + −sorted population play roles in epithelial polarity (STX2, STX3, EXOC6B) and adhesion (integrins ITGB1 and ITGB5, CLDN4, CDH1), cell migration (ADAMTS1 and ADAMTS15 metallopeptidases) and known markers of prostate cells (CD9, CD59, CD97, KRT18, KRT19). Conversely, the unsorted RNA is enriched in lymphocyte-specific transcripts and genes involved in antigen processing and presentation (several members of the HLA gene family, interleukins 10 and 16, CD74).

To further demonstrate that sequencing results from PACS-sorted RNA are similar to those obtained from RNA isolated directly from cells, we compared the transcriptional profiles of pure PC3 populations or those from VIM^+^-sorted cells to the profile of pure Raji cells. We identified 4,686 and 4,627 genes as differentially expressed (FDR ≤ 0.05) in the VIM + −sorted and the PC3 cell populations, respectively. Notably, 78 % of the genes found to be differentially expressed in the pure PC3 cells were also identified as differentially expressed from the VIM + −sorted material (Fig. 4b). These results support the use of PACS for high-dimensional gene expression profiling on sorted cell subtypes.

### Validation of differentially expressed genes with single-cell RT-PCR

**Fig. 5:**
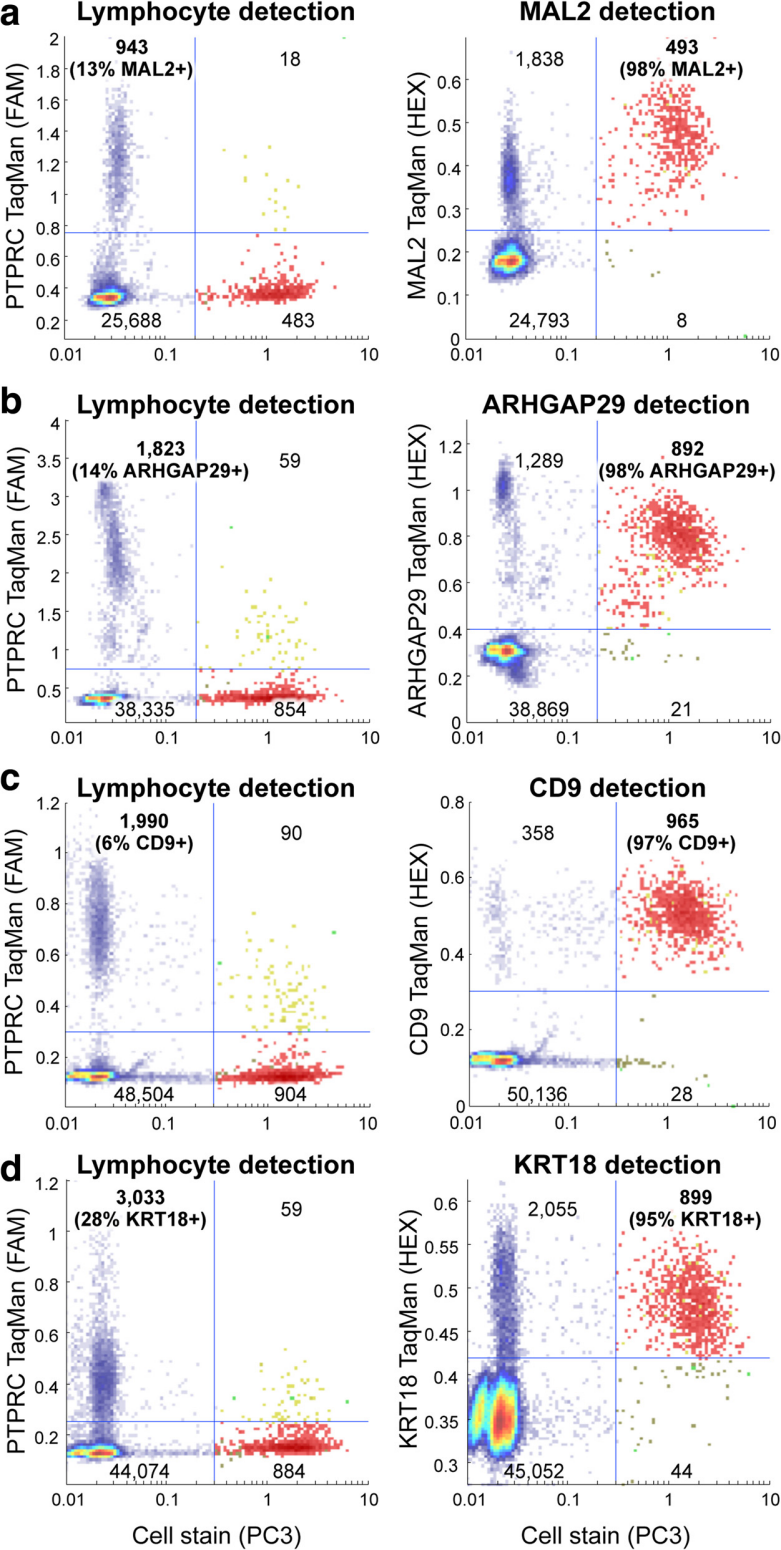
Confirmation of differentially expressed genes. **a**-**d** In the scatter plots, most drops containing PC3 cells (red) show expression of MAL2, ARHGAP29, CD9, and KRT18 (**a**-**d**, right panels), compared with sparse expression of the same genes in drops containing Raji lymphocytes (**a**-**d**, left panels) in multiplexed TaqMan assays. The blue lines are the thresholds to define clusters; the heat map colors are proportional to drop counts

We next sought to validate our transcriptome profiling results by investigating the single-cell expression profiles of selected genes (MAL2, ARHGAP29, CD9, and KRT18) found to be enriched in VIM+ PACS sorted RNA-Seq libraries (average log_2_ fold change 2.8 ± 0.23, Fig. 4a). Similar to previous experiments, PC3 cells were stained with calcein violet and spiked into a Raji background population. If the target transcripts were differentially expressed in PC3 cells, we would expect the TaqMan signal to be highly correlated with cell staining. As shown in Fig. 5, the majority of PC3 cells expressed each target gene (detection rates ranged between 95 %–98 %, mean = 97 ± 1 %), while only a small fraction of Raji cells did (between 6 %–28 % of cells, mean = 15 ± 5 %). The presence of target transcripts in some of the Raji population does not hinder PACS enrichment of PC3 cells, since the Raji cells are also positive for PTPRC expression detected with the multiplexed TaqMan approach. Subsequent to the above experiments, we added Cy5 dye detection capability to the PACS platform for expanded multiplex detection using calcein, a TaqMan assay targeting lymphocytes (PTPRC) and two TaqMan assays specific for prostate cancer cells (VIM and EPCAM, Additional file 4: Figure S4). These experiments validate the results from our transcriptome profiling data and underscore the value of PACS for rare cell subtype characterization and biomarker discovery.

## Discussion

PACS is a powerful approach to cell subtype identification, sorting and characterization. A fundamental feature of the PACS workflow is the use of inexpensive, reliable and readily available TaqMan assays. These assays allow for extremely sensitive and specific detection of nucleic acids and give PACS key advantages over other “top down” approaches to cell identification and sorting. We showed that a VIM+/PTPRC- multiplex TaqMan assay run on the PACS workflow enables high-fidelity identification of prostate cancer cells from both a background cell line or primary cell population. PACS is currently configured for detection of four fluorescent channels. With further expansion of our fluorescence detection capability, PACS should enable efficient multiplexing of at least five independent TaqMan assays using readily available probe dye and quencher combinations. The ability to multiplex both positive and negative selection markers with PACS provides efficient target cell identification with an extremely low false positive rate. It is important to note that PACS is flexible and not restricted to assaying only calcein viability dye and mRNA transcripts within cells. PACS has also been used to identify cell subtypes based on non-coding RNA and genomic DNA sequences, a capability not possible with single-cell barcoding methods that rely on polyadenylated mRNA [11, 12, 15]. Moreover, the PACS method can simultaneously interrogate cells labeled with fluorescent antibodies against surface markers together with TaqMan reaction fluorescence. This ability to correlate protein and nucleic acid biomarkers in a single workflow could prove highly effective for the unambiguous identification of many cell subtypes.

While PACS relies on preselected TaqMan assays to initially identify cell subtypes, unbiased transcriptome profiling on the contents of isolated cells can reveal essential information on the biology underlying the selected subpopulation. The principal challenge to expression profiling on PACS sorted material is the fragmentation of cellular RNA during droplet thermocycling in the presence of divalent cations, which produces RNA quality similar to FFPE samples. To overcome this challenge, we optimized a random-priming RNA library preparation protocol that works well on PACS-sorted RNA. The ability of this protocol to deliver accurate and minimally biased transcriptome information is evidenced by our data. We show that sequencing of PACS sorted RNA is sufficient to correctly differentiate among cell subtypes and extract cell-specific transcriptional signatures. In addition to transcriptome profiling, PACS could also enable full genome sequencing on isolated cell subtypes. We previously demonstrated the ability to perform targeted genomic DNA sequencing with PACS and a related droplet-based enrichment method, MESA [15, 40]. The combination of transcriptome and genome sequencing makes PACS a unique platform to reveal not only key aspects of cell behaviors, but also the genetic drivers responsible for those behaviors.

The potential applications of PACS are numerous and encompass multiple fields in the life sciences. With a demonstrated throughput of over 100,000 cells per experiment [15], PACS is uniquely suited for analyzing large heterogeneous cell populations, including immune cells, disaggregated tumors and even circulating tumor and fetal cells, especially when combined with cell pre-enrichment or depletion strategies. Moreover, PACS is most useful when the cell subtype can’t be identified with antibodies, either because a suitably specific antibody is not available or the biomarker of interest is not translated into a protein. For example, PACS could allow the detection and isolation of human cells latently infected with HIV by directly targeting the genome integrated virus with TaqMan reactions [35]. Subsequent transcriptome profiling on these isolated cell reservoirs could identify critical host cell factors contributing to persistent infection and/or latency. Another unique PACS use case would be for the detection of non-coding RNAs or alternative splicing events that mark a disease state or contribute to its pathology [47].

## Conclusions

PACS is a novel cytometry method that is capable of detecting and isolating target cell subtypes with high sensitivity. The throughput, minimal sample processing and use of readily available TaqMan assays afford PACS significant advantages over existing tools for studying cellular heterogeneity. With the added ability to sequence RNA following sorting, we anticipate PACS will prove widely useful for enumerating and transcriptionally profiling rare cell subtypes from complex biological samples. Understanding the transcriptional status of these rare cells will aid in the study of human health and the causes of disease.

## Methods

### Cell culture and staining

Human PC3 and DU145 prostate cancer and Raji B-lymphocyte cell lines (publicly available, ATCC catalog numbers: PC3 CRL-1435, DU145 HTB-81 and Raji CCL-86) were cultured in complete DMEM (DMEM with 10 % FBS, 100 U/ml penicillin, and 100 μg/ml streptomycin) at 37 °C with 5 % CO_2_. Before cell staining, adherent PC3 and DU145 cells were detached with 0.25 % trypsin-EDTA (Invitrogen). Cells were then pelleted at 400 g for 4 min and washed once in Phosphate Buffered Saline solution (PBS, Life Technologies). Cells were resuspended in 1 ml Hank’s Balanced Salt Solution (HBSS, Life Technologies) with 5 μM Calcein Violet AM (eBioscience) and stained for 20–30 min at room temperature in the dark. Cells were then washed once with HBSS and resuspended in PBS that was density matched with OptiPrep (Sigma-Aldrich) prior to encapsulation in microfluidic droplets. For spike-in experiments, 5 μl aliquots of cell suspension were combined with an equal amount of trypan blue (Life Technologies), then loaded on chamber slides and counted with the Countess Automated Cell Counter (Invitrogen).

### Peripheral blood mononuclear cell (PBMC) isolation

6 ml of whole blood was mixed with an equal volume of PBS with 2 % FBS (Invitrogen), then loaded on a Histopaque-Accuspin column (Sigma). The column was centrifuged at 1,000 RCF for 10 min, and the plasma and buffy coat layers loaded on an Acrodisc WBC (Pall). The filter was washed twice with 10 ml PBS, and leukocytes were eluted in 7 ml of PBS-2 % FBS. Cells were pelleted at 200 g for 10 min and resuspended in PBS-OptiPrep as previously described.

### Microdroplet TaqMan RT-PCR

TaqMan reaction primers and probes were purchased as a pre-mixed assay from Integrated DNA Technologies (IDT). Amplification primers for the VIM and PTPRC genes were previously described [15, 16]. Exon junctions targeted by the IDT TaqMan assays are as follows: ARHGAP29 ex. 20–21, CD9 ex. 2–3, EPCAM ex. 1–2, GAPDH ex. 7–8, KRT18 ex. 4–5, KRT19 ex. 1–3, MAL2 ex. 3–4. SuperScript III Reverse Transcriptase (Invitrogen) and Platinum Multiplex PCR Master Mix (Applied Biosystems) were used for the microdroplet single-cell TaqMan reactions with the following thermocycling conditions: 15 min 50 °C, 93 °C for 2 min, 35–40 cycles of 92 °C for 15 s and 60 °C for 1 min. Reverse transcription in the droplets was performed only on transcripts targeted by the TaqMan assays, not on the whole transcriptome. For detection and sorting, thermocycled droplets were transferred to a 1 ml syringe and reinjected into a microfluidic device.

### Fabrication and operation of microfluidic devices

We performed the microfluidic droplet handling on devices made from polydimethylsiloxane (PDMS) molds bonded to glass slides; the device channels were treated with Aquapel to make them hydrophobic. The PDMS molds were formed from silicon wafer masters with photolithographically patterned SU-8 (Microchem) on them. We operated the devices with syringe pumps (NewEra), which drove cell suspensions, reagents and fluorinated oils (Novec 7500 and FC-40) with 5 % PEG-PFPE block-copolymer surfactant into the devices through polyethylene tubing [48].

Droplet fluorescence detection was carried out on a inverted microscope (Motic) using four coincident lasers (405 nm, 473 nm, 532 nm, and 640 nm, CNI lasers) to excite the cell viability stain and TaqMan probes. The resulting fluorescence channels (centered at 440 nm, 510 nm, 572 nm, and 680 nm) were separated from the lasers and each other with dichroic filters (Semrock) before being detected by four PMTs (Thorlabs). The detection was processed in real time using LabVIEW and an FPGA card (National Instruments). For sorting, the FPGA card was programmed to activate a high voltage power supply (Trek) connected to a salt-water electrode on the device to dielectrophoretically sort any drop that matched the desired fluorescence profile [49]. The drop scatter plots and statistics were generated using MATLAB. In the PACS scatterplots (e.g., Fig. 2a), the vertically oriented calcein violet threshold was set manually in the empty gap just to the left of the tightly clustered, brightly stained drops (red points). The thresholds for the TaqMan probes were set by placing an upper bound on the “dark” cluster on the lower left of each cell stain vs. probe plot (solid, multicolored regions); those thresholds were chosen to sit in the middle of the flat shoulder that was attached to the top of the dark cluster. Rgl was used for the 3D plots in Additional file 4: Figure S4c [50].

### Quantitative RT-PCR analysis of PACS-sorted RNA

Emulsions were broken as previously described [15]. The aqueous fraction from the droplets was diluted with water and split into two TaqMan RT-PCR assays targeting either KRT19 or GAPDH. qRT-PCR reactions were performed on the AriaMX Real-Time PCR System (Agilent Technologies). Differences in expression levels were calculated using background normalized Ct values from qRT-PCR amplification curves. For the KRT19 enrichment data in Fig. 3c, all Ct values were normalized using GAPDH as a standard. Prism software was used for the amplification plots in Fig. 3.

### RNA recovery and sequencing library preparation

Positively sorted TaqMan emulsions were broken using perfluoro-1-octanol and the aqueous fraction was diluted in water. Total RNA was purified using the Quick-RNA Microprep kit (Zymo), performing on column DNA digestion with 5 μl DNAse I and 5 μl Exonuclease I (NEB) for 90 min at RT to decrease genomic and TaqMan PCR amplicon contamination in the downstream preparation steps. The RNA was recovered by performing two 8-μl elutions. After rRNA depletion (Ribo-Zero Gold kit, Illumina), libraries were prepared using the SMARTer Stranded RNA-Seq kit (Clontech) and amplified using 15 PCR cycles. The libraries were purified with Select-a-Size DNA Clean & Concentrator columns (Zymo) with a 75 bp cutoff, and eluted in 22 μl. Libraries were analyzed on a High Sensitivity DNA Assay chip with a Bioanalyzer (Agilent Technologies), and sequenced on a HiSeq4000 in single-end 50 bp multiplexed runs. Sequenced reads passing quality control (FastQC, cutadapt, trimmomatic, [51, 52]) were aligned to the hg19 human transcriptome (iGenomes) using TopHat2-Bowtie2 mappers [53, 54]. Downstream analyses were performed using samtools, HTSeq [55], and the edgeR and gplots packages for R [56, 57]. The *Benjamini–Hochberg* procedure was used to control for multiple comparisons.

### Ethics

Anonymous blood samples were initially obtained from a commercial provider (AllCells) that operates with independent IRB approval. Donor anonymity was protected using United States HIPAA privacy and security rules. Following NIH policies governing human sample use, further ethical approval was not required for this study.

### Consent to publish

Consent was obtained by the commercial blood supplier at the time of sample donation.

### Availability of data and materials

The raw data and gene counts are accessible through the Gene Expression Omnibus (GEO) database at the following address: http://www.ncbi.nlm.nih.gov/geo/query/acc.cgi?acc=GSE80551.

## Additional files

Additional file 1: Figure S1.
*PACS detection from a primary cell population.* (a-b) PC3 spiked in Peripheral Blood Mononuclear Cells (PBMC) can be detected and sorted based on multiplex TaqMan assays. Scatter plots show cell stain (x-axis) versus PTPRC (left panels) and EPCAM (a) or ARHGAP29 (b) fluorescence (right panels). Red dots represent droplets with PC3 cells. The blue lines are the thresholds to define clusters; the heat map colors are proportional to drop counts. (TIF 8059 kb) Additional file 2: Figure S2.
*Distribution of fold changes of gene expression.* (a) Histogram representing the distribution of log_2_-fold change of genes expressed in PACS VIM + −sorted material and the heterogeneous Raji:PC3 (10:1) population (white bars). Red bars show the distribution exclusively for the genes differentially expressed between the two samples. (Insert) Box plot of the log_2_-fold change distribution for the differentially expressed genes in (a). (TIF 7680 kb) Additional file 3: Figure S3.
*Correlation of read counts*. The scatter plots show the correlation between read counts in VIM + −sorted material and PC3 cells (a) and the correlation between read counts in the heterogeneous Raji:PC3 population and Raji cells (b) for the differentially expressed genes in Fig. 4a. The red line is a linear fit to the data. ρ indicates the Pearson’s correlation coefficient. The data is plotted as log_2_ of the average read count (in counts per million, CPM) normalized for library size. The green data points in (a) represent a subset of the 23 % (1057) of differentially expressed genes that were unique to the VIM+ PACS sort vs. pure Raji comparison from the Fig. 4b Venn diagram. The blue data points were also identified exclusively in the pure PC3 vs. pure Raji comparison (998 genes shown in Fig. 4b). (TIF 14209 kb) Additional file 4: Figure S4.
*PACS workflow showing 4-channel multiplex detection of calcein stained PC3 and Raji cells.* In many biological samples, it isn’t possible to specifically stain one cell type. Therefore, we performed PACS on a heterogeneous population of Raji:PC3 cells (10:1 ratio), staining both target and background cell populations. In this experiment, we also employed a third TaqMan assay targeting EPCAM. (a) A mixed population of calcein violet-stained Raji and PC3 cells can be separated as a PTPRC+ (green dots) and a PTPRC−/EPCAM+/VIM+ cluster (red dots), respectively. (b) In the absence of PC3 cells, the PTPRC−/EPCAM+/VIM+ cluster is absent and there is minimal detection of false positive Raji cells. The blue lines are the thresholds to define clusters; the heat map colors are proportional to drop counts. (c) The calcein-violet positive drops from (a) and (b) are represented in 3D plots. The plots highlight the position of the PC3 (red) and Raji (green) clusters in the fluorescent space for the heterogenous Raji:PC3 population (left) or Raji only cells (right). The black cluster represents calcein positive drops with no TaqMan fluorescent signal. The blue dots represent drops that are positive for all TaqMan assays (38, left panel). This data demonstrates the utility of the TaqMan multiplexing approach to accurately identify target cells without relying on cell-type specific staining. (TIF 19652 kb)

## Abbreviations

PCR: polymerase chain reaction
RT: reverse transcription
PACS: pcr-activated cell sorting
RNA: ribonucleic acid
DNA: deoxyribonucleic acid
FACS: fluorescence-activated cell sorting
FISH-FC: fluorescence in situ hybridization-flow cytometry
NIH: National Institutes of Health
