# Supplementary figures and images for: RNA-Seq following PCR-based sorting reveals rare cell transcriptional signatures
Source: BMC Genomics. 2016 May 17;17:361. doi: 10.1186/s12864-016-2694-2 (PMC4869385; doi:10.1186/s12864-016-2694-2)

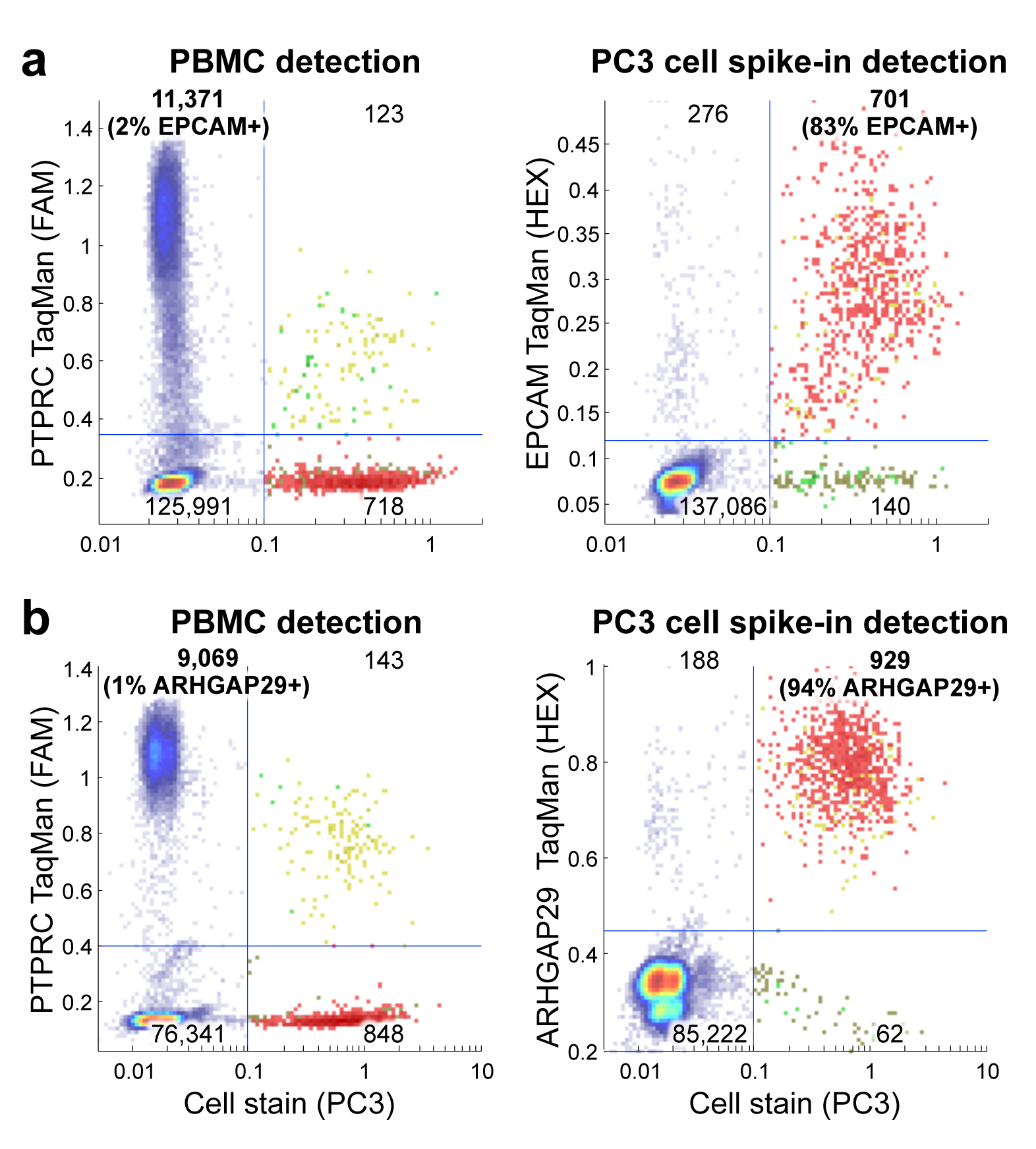

Supplement: Additional file 1: Figure S1. — PACS detection from a primary cell population. (a-b) PC3 spiked in Peripheral Blood Mononuclear Cells (PBMC) can be detected and sorted based on multiplex TaqMan assays. Scatter plots show cell stain (x-axis) versus PTPRC (left panels) and EPCAM (a) or ARHGAP29 (b) fluorescence (right panels). Red dots represent droplets with PC3 cells. The blue lines are the thresholds to define clusters; the heat map colors are proportional to drop counts. (TIF 8059 kb) [file 12864_2016_2694_MOESM1_ESM.tif]

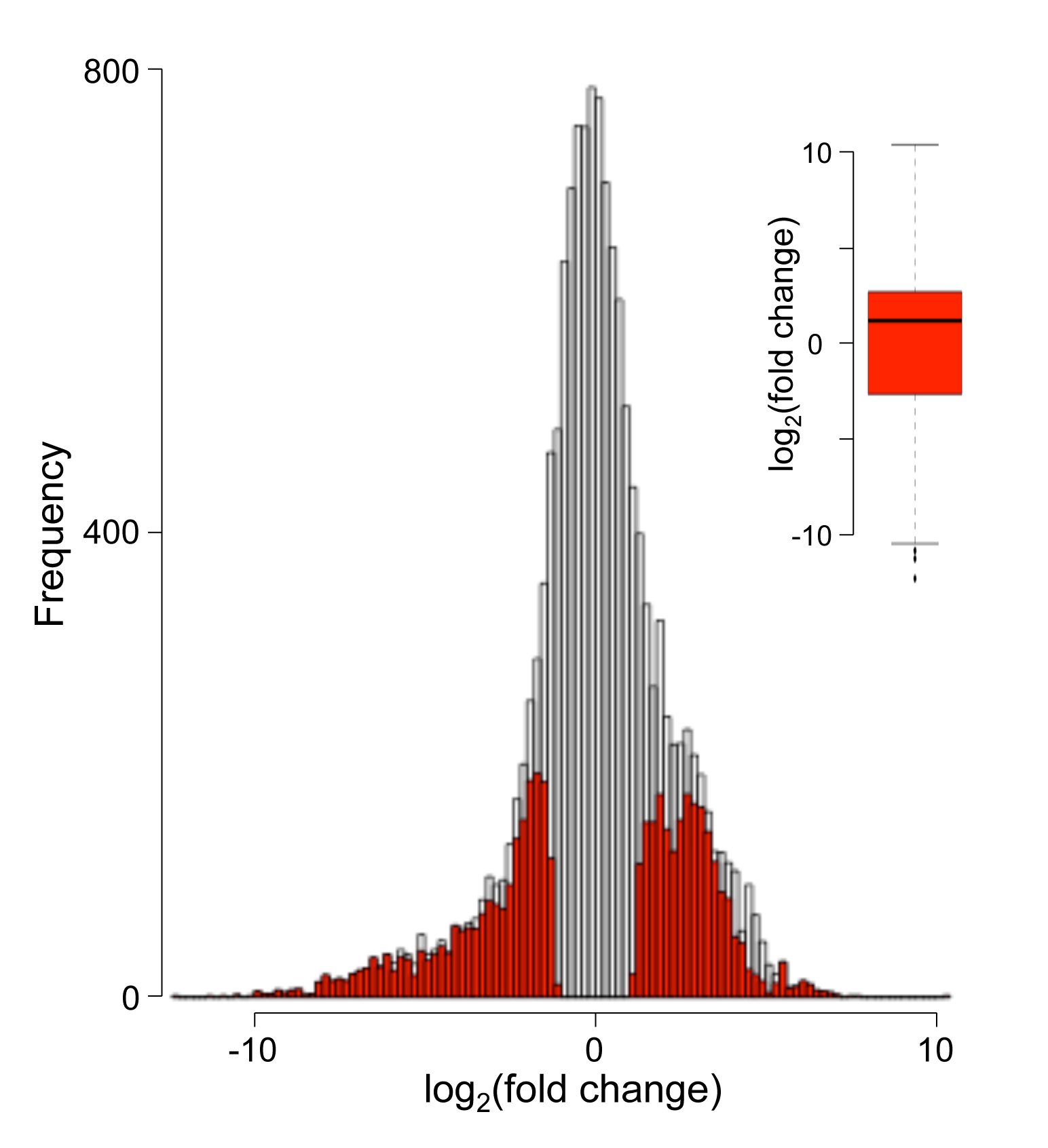

Supplement: Additional file 2: Figure S2. — Distribution of fold changes of gene expression. (a) Histogram representing the distribution of log2-fold change of genes expressed in PACS VIM + −sorted material and the heterogeneous Raji:PC3 (10:1) population (white bars). Red bars show the distribution exclusively for the genes differentially expressed between the two samples. (Insert) Box plot of the log2-fold change distribution for the differentially expressed genes in (a). (TIF 7680 kb) [file 12864_2016_2694_MOESM2_ESM.tif]

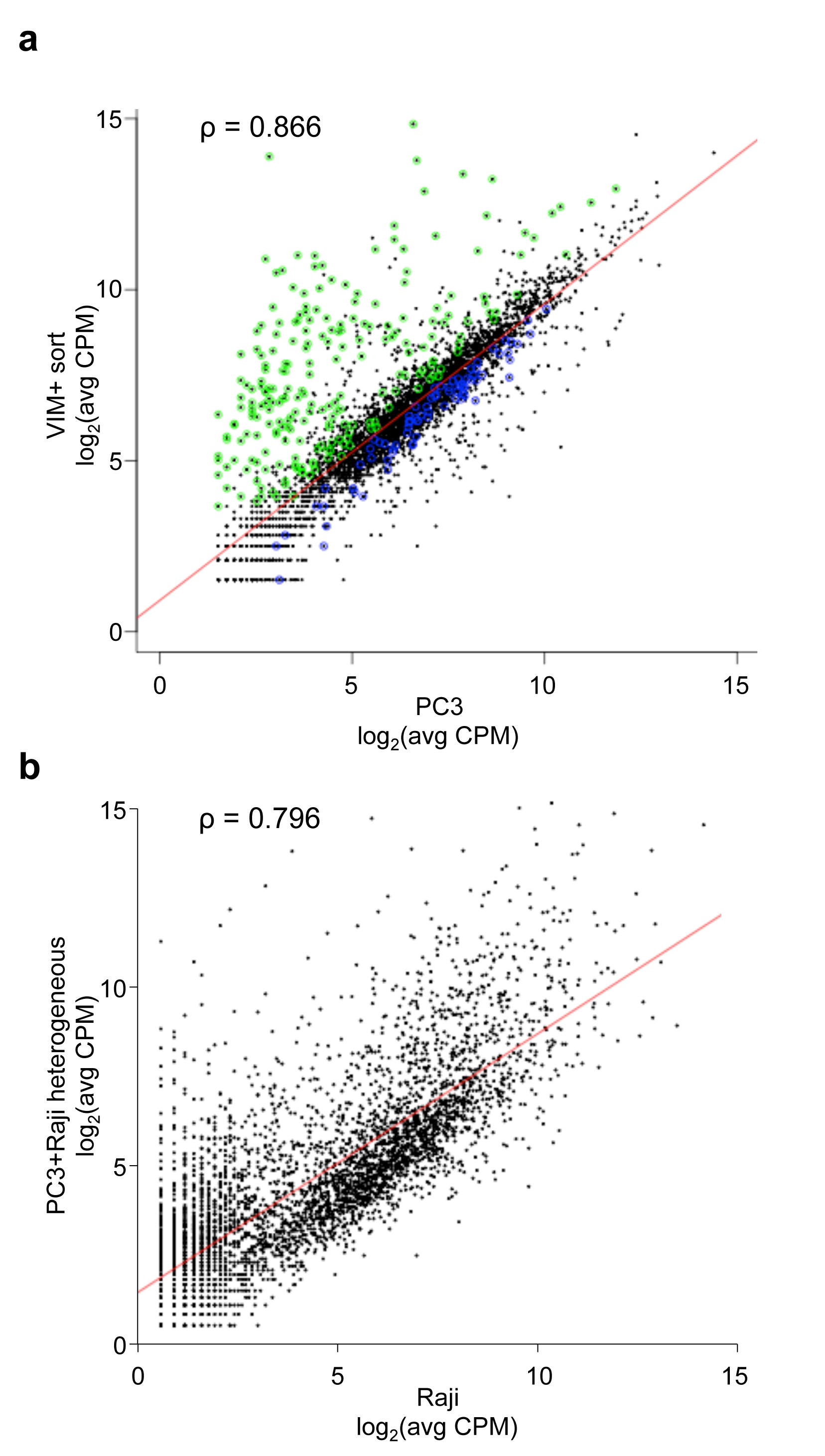

Supplement: Additional file 3: Figure S3. — Correlation of read counts. The scatter plots show the correlation between read counts in VIM + −sorted material and PC3 cells (a) and the correlation between read counts in the heterogeneous Raji:PC3 population and Raji cells (b) for the differentially expressed genes in Fig. 4a. The red line is a linear fit to the data. ρ indicates the Pearson’s correlation coefficient. The data is plotted as log2 of the average read count (in counts per million, CPM) normalized for library size. The green data points in (a) represent a subset of the 23 % (1057) of differentially expressed genes that were unique to the VIM+ PACS sort vs. pure Raji comparison from the Fig. 4b Venn diagram. The blue data points were also identified exclusively in the pure PC3 vs. pure Raji comparison (998 genes shown in Fig. 4b). (TIF 14209 kb) [file 12864_2016_2694_MOESM3_ESM.tif]

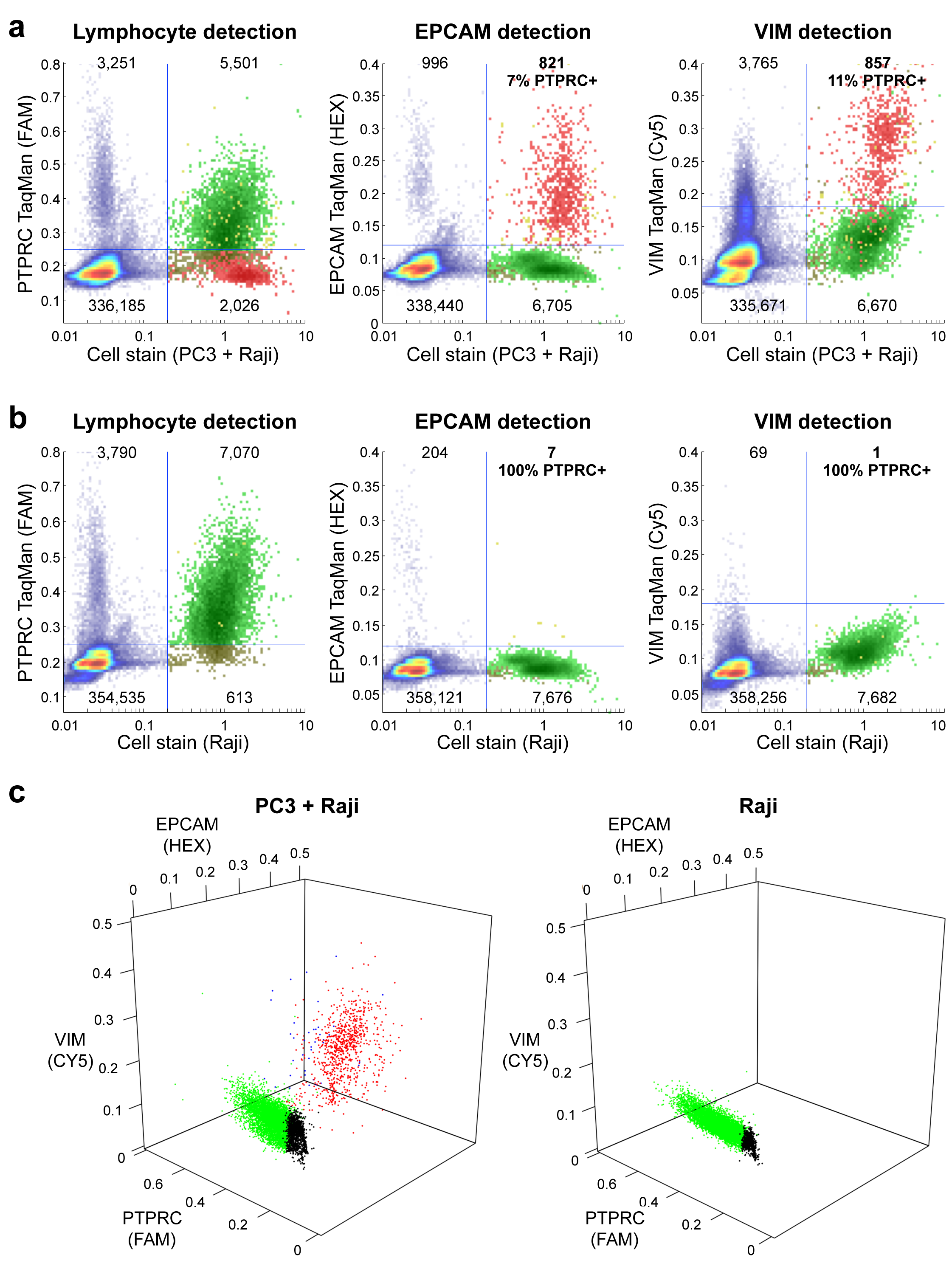

Supplement: Additional file 4: Figure S4. — PACS workflow showing 4-channel multiplex detection of calcein stained PC3 and Raji cells. In many biological samples, it isn’t possible to specifically stain one cell type. Therefore, we performed PACS on a heterogeneous population of Raji:PC3 cells (10:1 ratio), staining both target and background cell populations. In this experiment, we also employed a third TaqMan assay targeting EPCAM. (a) A mixed population of calcein violet-stained Raji and PC3 cells can be separated as a PTPRC+ (green dots) and a PTPRC−/EPCAM+/VIM+ cluster (red dots), respectively. (b) In the absence of PC3 cells, the PTPRC−/EPCAM+/VIM+ cluster is absent and there is minimal detection of false positive Raji cells. The blue lines are the thresholds to define clusters; the heat map colors are proportional to drop counts. (c) The calcein-violet positive drops from (a) and (b) are represented in 3D plots. The plots highlight the position of the PC3 (red) and Raji (green) clusters in the fluorescent space for the heterogenous Raji:PC3 population (left) or Raji only cells (right). The black cluster represents calcein positive drops with no TaqMan fluorescent signal. The blue dots represent drops that are positive for all TaqMan assays (38, left panel). This data demonstrates the utility of the TaqMan multiplexing approach to accurately identify target cells without relying on cell-type specific staining. (TIF 19652 kb) [file 12864_2016_2694_MOESM4_ESM.tif]
